# Representations of mpox in UK news media during the 2022 outbreak: a qualitative analysis

**DOI:** 10.1186/s12889-026-28060-2

**Published:** 2026-06-19

**Authors:** Marthe Le Prevost, Emily Jay Nicholls, Davide Bilardi, Alexandra David, Sailesh Kandasamy, Marco Stizioli, Sarah Denford, Tom May, Emily Tan, Shema Tariq

**Affiliations:** 1 UCL Innovative Clinical Trials Unit, Institute of Clinical Trials & Methodology, University College London, London, UK; 2 Institute of Global Health, University College London, London, UK; 3 Fondazione Penta ETS, Padua, Italy; 4 Nuffield Department of Primary Care Health Sciences, University of Oxford, Oxford, UK; 5 University College London, London, UK; 6 European AIDS Treatment Group, Brussels, Belgium; 7 NIHR Health Protection Research Unit in Evaluation and Behavioural Science, Population Health Sciences, Bristol Medical School, University of Bristol, Bristol, UK

**Keywords:** Mpox, Monkeypox, News media, United Kingdom, Qualitative research

## Abstract

**Background:**

The 2022 global mpox outbreak was the first to involve sustained community transmission outside endemic regions, disproportionately affecting gay, bisexual and other men who have sex with men (GBMSM). News media plays a critical role during outbreaks by disseminating information, shaping public perception and influencing health protection behaviours. This qualitative study examined how UK news media represented mpox during the 2022 outbreak, with a particular focus on public health messaging and the discursive framing of affected communities.

**Methods:**

Using the NexisUK® database, we retrieved UK print and online articles mentioning “mpox”, “monkeypox”, or “monkey pox” in headlines, published between May and December 2022. Five national newspapers were selected based on readership and political stance (The Times/Sunday Times, The Guardian, The Sun, The Daily Mail/Sunday Mail and the Daily Mirror/Sunday Mirror). Following deduplication and screening, we applied a rotating weekday sampling frame and analysed articles thematically in NVivo.

**Results:**

We identified 746 articles in the five selected newspapers between May and December 2022. Coverage peaked in May 2022 (*n* = 223 articles) and then waned, despite incident cases rising in June (*n* = 1,185) and July (*n* = 1,453). We analysed 79 sampled articles. Key themes included communication of mpox characteristics, delivery of health promotion, and ‘anchoring’ mpox to other infectious diseases (e.g. COVID-19, chickenpox) to aid understanding. Articles described mpox’s epidemiological origins in Africa, transmission routes, and epidemiological updates, often noting transmission during GBMSM events. Public health messaging focused on advice about transmission reduction, access to care/prevention services, and vaccine availability (including concerns over supply). GBMSM were frequently depicted as being at high risk, sometimes drawing upon stereotypes and language that potentially reinforced stigma. Notably, few articles included perspectives from individuals with lived experience of mpox.

**Conclusion:**

UK news media interest in mpox peaked early in the outbreak and waned despite rising cases. News media played a vital role in disseminating information and public health messaging (often drawing parallels with other familiar infections) but the framing of GBMSM may also have reinforced stigma. The absence of personal testimonies represents a missed opportunity for inclusive messaging. Future outbreak reporting should involve affected communities to co-produce and promote accurate, non-stigmatising communication.

**Supplementary Information:**

The online version contains supplementary material available at 10.1186/s12889-026-28060-2.

## Introduction

Mpox (previously known as monkeypox), a zoonotic disease first identified in 1958, has been endemic in several countries across Central and West Africa since 1970, and remains a significant public health threat today, especially in the Democratic Republic of the Congo (DRC) [1]. In May 2022, an outbreak of mpox driven by a new Clade IIb strain emerged in previously non-endemic countries, particularly in Europe and North America [2]. The 2022 outbreak marked the first instance of sustained community transmission of mpox without travel links to endemic countries and was declared a “Public Health Emergency of International Concern” (PHEIC) by the World Health Organization (WHO) in July 2022 [3].

The first mpox case in the United Kingdom (UK) was reported on 6 May 2022. By 31 December 2022, there were 3,732 confirmed and highly probable cases, with the peak incidence occurring in mid-July, when 1,399 cases were recorded [4]. This outbreak almost exclusively affected networks of gay, bisexual, and other men who have sex with men (GBMSM), with sexual contact the primary mode of transmission [4–6]. Prior to this outbreak, only seven mpox cases had been reported in the UK. Of these, four were among people with a recent history of travel to endemic countries, two were household contacts of cases, and one was a healthcare worker involved in the care of an infected patient [7]. Despite a decline in cases since July 2022, new cases continue to be reported in the UK; between 1 and 41 cases were reported per month between 2023 and 2025 (up to 31 July 2025) [7]. During this period, 2023–2025, a total of 572 cases of Clade IIb mpox were reported, comprising a mix of infections acquired both within the UK and abroad [7]. The decline in mpox transmission in the UK in the summer of 2022 is believed to be primarily due to behaviour changes among GBMSM communities, such as reducing sexual partner numbers [8]. In addition, the use of modified vaccinia Ankara – Bavarian Nordic (MVA-BN) vaccine has been demonstrated to reduce the risk of acquisition of mpox, as well as severity of disease, and contributed further to sustained low incidence in the UK [8].

Public health measures to limit transmission of mpox include contact tracing, isolating individuals suspected or confirmed to have mpox and their close contacts, and the MVA-BN vaccine [9]. In the UK, the vaccination strategy targeted GBMSM reporting multiple sexual partners and/or group sex, staff who worked in sex on premises venues, close contacts of individuals with confirmed mpox, and healthcare professionals at risk of occupational exposure [10]. Communicating these measures clearly to affected communities and the wider public was therefore a central component of the response.

Traditional news media (which we define as print and digital newspaper articles providing news, commentary, and analysis on current events) plays a vital role during infectious disease outbreaks by disseminating information, shaping public perceptions, and influencing the perceived severity of the situation and broader public discourse [11]. Emerging as COVID-19 was still having global impact, the mpox outbreak garnered significant media coverage. In a UK survey conducted between 15 June and 27 July 2022 via community social media channels and the Grindr dating app, 27% of 1,932 respondents identified newspapers as one of the top three sources where they had heard about mpox [12]. This role of news media becomes particularly consequential during pandemics, when policymaking is rapid, evidence is evolving, and news media act as a key conduit linking science, public health authorities, policymakers, and the public [13].

However, news media are not neutral actors. Differences in scientific quality, source selection, and framing can shape public attitudes and behaviours, with important implications for public health responses and wider societal attitudes [13, 14]. A critical discourse analyses of HIV reporting in Ireland between 2006 and 2016 identified moralising narratives around sexuality and personal responsibility, with consequences on stigma, service access, and policy priorities [15]. More recently, research on COVID 19 has demonstrated how media framing around the origins of the virus had “downstream effects” on public beliefs and willingness to accept public health advice, while labels such as the “Chinese virus” also amplified blame directed at China and contributed to prejudice against Chinese and Asian Americans [16].

Against this backdrop, news media analysis has been widely used in relation to public health issues, including within sexual health. Studies on HPV vaccine coverage and COVID-19, for example, have predominantly used quantitative methods such as content (systematic study of communication content to identify patterns or meanings) [17–20] and frequency (how often certain words, topics, or themes appear) [14, 21, 22] analysis, while qualitative approaches such as critical discourse analysis (how language and framing shape power, ideology, and public perception) [15, 23] and thematic analysis [24] have been used to explore how media narratives shape public understanding of HIV and reinforce or challenge dominant health agendas.

To date, few studies have analysed newspaper coverage of the 2022 mpox outbreak, and those that have either focused on English language news media more broadly rather than UK specifically [25], or have focused on tabloid and right-leaning media outlets [26]. This paper addresses these gaps through a qualitative analysis of UK news media coverage, drawing on a range of outlets spanning the political spectrum, with a particular focus on public health messaging, and the discursive framing of affected communities. By including broadsheets and left-leaning outlets, alongside tabloids and right-leaning media, we aimed to capture how mpox was constructed across the spectrum of the media landscape, highlighting shared narratives and key differences in how risk and responsibility were described. In an increasingly polarised discursive space, we hypothesised that diverse sampling was essential in gaining a more comprehensive view of news media representations of mpox.

## Methods

This is a qualitative study, comprising thematic analysis of UK news media coverage of mpox between May and December 2022, based on articles identified using headline search terms and subsequently coded with a structured codebook before developing broader themes using a Codebook Thematic approach [27]. A codebook thematic approach integrates inductive coding with a collaboratively developed and iteratively refined codebook to ensure consistency across the research team, with themes generated through systematic comparison and regular team discussions to strengthen analytic rigour. This work is part of the larger VERDIQual study, a multimethod qualitative investigation of mpox in Italy, Nigeria, Thailand and the UK [28], undertaken within the international VERDI Consortium [29].

### Terminology

In this article, we use the term “gay, bisexual and men who have sex with men (GBMSM)” as an inclusive umbrella term for men who may be affected by the issues under study but do not necessarily identify as gay or bisexual. When presenting or interpreting news media material, we retain the terms used in those sources (e.g. “gay men”, “gay and bisexual men”) to avoid retrospectively applying GBMSM in ways that might misrepresent the language or focus of the media content.

In addition, our data are UK national newspaper articles (print and digital editions), and we use the term “newspapers” when reporting our sampling and results, reserving the broader term “news media” when referring to the wider role of these outlets in shaping public perceptions and public health messaging.

### Dataset

On the 15th of November 2023, we searched for UK print and online articles relating to mpox on NexisUK ®, a continuously updated online database of international, national and regional news sources. We searched content from five national weekday and Sunday newspapers published between 1 st May and 31 st December 2022, corresponding to the rise, peak and fall of mpox cases in the UK [4]. We used the search terms “mpox” OR “monkeypox” OR “monkey pox”, limited to headlines. Searches were limited to headline mentions of the search terms in NexisUK ® to capture articles in which mpox was the central focus and for reasons of feasibility. The five newspapers included in the search were: The Times/Sunday Times, The Guardian, The Sun, The Daily/Sunday Mail, and The Mirror/Sunday Mirror. These all have print and online versions and were selected due to their wide readership, and diversity in political stance and format i.e. tabloid and broadsheet publications (Table 1).

**Table 1:** Characteristics of newspapers included in the analysis

Title	Format	Political Party supported in 2019 general election^*^	Year of latest circulation figures	Print Circulation (average per issue)
The Guardian	Broadsheet	Labour Party	2021^**^	108,687
The Daily Mail	Tabloid	Conservative Party	2025^***^	631,191
Mail on Sunday				534,119
The Daily Mirror	Tabloid	Labour Party	2025^***^	185,945
Sunday Mirror			2025^***^	185,145
The Sun	Tabloid	Conservative Party	2020**	1,250,634
The Times & Sunday Times	Broadsheet	Conservative Party	2020**	368,929

^*^Source: https://en.wikipedia.org/wiki/Endorsements_in_the_2019_United_Kingdom_general access November 2023 (NB this information was correct at the time of data collection but did change for the 2024 election)

^**^Source: https://en.wikipedia.org/wiki/List_of_newspapers_in_the_United_Kingdom_by_circulation (Data from Audit Bureau of Circulations), accessed August 2025

^***^Source: ABC Certified Products https://www.abc.org.uk/data/reports-and-certificates, accessed August 2025

The following data were extracted for each article using a standardised form in Excel: headline, date of publication, word count, newspaper title, and whether the article was available online, in print, or both. Two reviewers (EJN and MLP) undertook initial screening, excluding duplicates (same headline and word count, or same/similar headline but shorter version) and articles that did not cover mpox or did not discuss mpox in relation to the UK.

MLP initially coded a random selection of ~ 10%, identifying potential themes. Given the repetition of themes across this selection, we restricted further analysis to a sample of the eligible articles. We used an adapted version of the ‘Constructed Week Sampling’ method [30], in which all articles published on one day each week were sampled over the study timeframe. The selected day of the week varied, with the next consecutive day chosen each week to ensure the final sample represented all seven days of the week. A dataset comprising all articles would preclude in-depth qualitative analysis; sampling was essential for feasibility and rigour.

### Overview of UK news media coverage of mpox

**Fig. 1:**
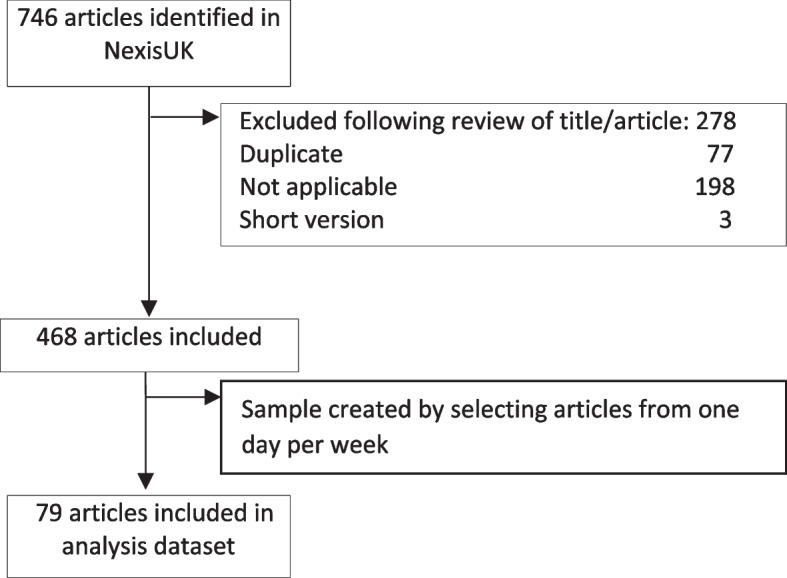
Flowchart of newspaper articles identified in the news media analysis

In total, 746 articles were identified in the initial NexisUK search; 278 were excluded following full text review (Fig. 1). The analysis sample (*n* = 79) was derived from the remaining 468 articles.

### Analysis

The selected articles were uploaded and analysed thematically in NVivo V.14. MLP undertook initial open coding of 20 articles, with double-coding by members of the study team (DB, EJN, ST) [14, 15], to generate a codebook. Following this, MLP analysed all sampled articles using the codebook and developed broader themes using a Codebook Thematic Analysis approach [27].

As part of the multi country qualitative VERDI study, each country team established a Community Advisory Board (CAB), including representatives from communities affected by mpox. CAB members reviewed study documents and contributed to the analysis and manuscripts. For this paper, analysis and insights were shared with co investigators and a CAB representative (with expertise by experience) until consensus was reached.”

## Results

**Fig. 2:**
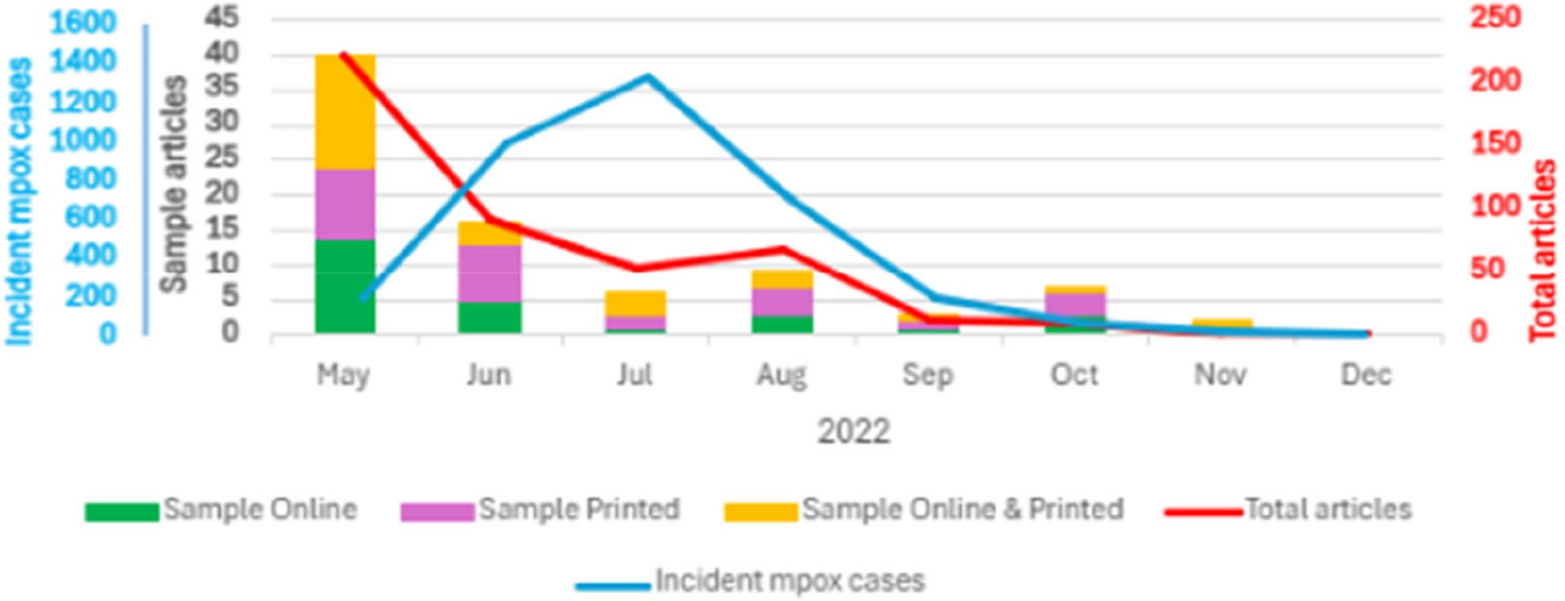
Total number of articles on mpox in five selected newspapers, number of articles sampled and incident number of mpox cases in the UK, May-December 2022

We identified 468 articles on mpox in the five included newspaper titles between 1 May and 31 December 2022, of which 79 were included in our analysis (Fig. 2). The analysed sample comprised 10 articles from The Guardian, 29 from the Daily Mail/Mail on Sunday, 20 from the Daily Mirror/Sunday Mirror, 12 from The Sun and 8 from The Times/Sunday Times. The highest number of articles was recorded in May 2022 (*n* = 223 of which 40 were included in the analysis sample), at the start of the outbreak. Coverage steadily declined from June 2022 onwards, to zero articles in December 2022. News coverage declined early in the outbreak, with an approximate 50% decrease in published articles between May and June 2022. This decline predated the reduction in mpox cases in the UK, which occurred after the peak of cases in July 2022 (Fig. 2) [31–36].

Our qualitative analysis of representations of mpox in news media focused on two key areas: (i) public health messaging and (ii) discursive framing of affected communities. From this analysis, we identified three overarching themes: mpox characteristics, delivery of health promotion and ‘Anchoring’ mpox to other infectious diseases (see Supplementary Table 1). These are now presented in further detail below.

### Mpox characteristics

This outbreak involved the emergence of a virus that was previously rare in the UK and other high-income settings. News media were therefore well-placed to communicate essential information to a readership with little knowledge of mpox. Factual information about mpox (covering both the virus and the disease) was the predominant subject of news media articles during the study period. Articles discussed the historical origins of mpox, describing its discovery in laboratory monkeys in 1958, its identification in humans in the DRC in 1970, and how it had subsequently established itself as an endemic infection in Central and Western Africa, with many articles reporting it as a zoonosis. Early in the outbreak, newspapers reflected concerns about the risk of endemicity, should mpox spread to pets or wildlife:

“A spill-over event could potentially lead to the virus establishing in European wildlife and the disease becoming an endemic zoonosis.”

(Unnamed source at the European Centre for Disease Prevention and Control, quoted in Mail Online 24/05/2022)

Newspapers speculated on the origins of the then emerging outbreak in the UK and other high-income countries; reports early in the outbreak already linked the outbreak to venues and events connected to the gay community:

“Outbreaks have been traced to a gay sauna in Madrid and a Gran Canaria pride festival attended by 80,000 people from Britain and other European countries”

(Mail Online, 24/05/22)

The urgency of the unfolding outbreak was reinforced by news media reporting of official warnings by organisations such as the WHO, and the later declaration of mpox as a PHEIC, described by The Guardian as “*the strongest call to action the agency can make*” (23/07/22). Yet, in the context of global uncertainty about the pandemic potential of mpox, and the need to galvanise prevention efforts, articles also reported officials’ belief that further spread could be averted through vigilance and concerted action:

“[The mpox outbreak] is preventable [but] we can’t take our eye off the ball on what’s happening.”

(Maria Van Kerkhove, World Health Organization, quoted in Mail Online 24/05/2022)

“The risk of the disease [mpox] becoming established in these countries [is] real but preventable at this point.”

(Tedros Ghebreyesus, Director General of the World Health Organization, quoted in The Sun, 09/06/2022).

As cases increased in the UK and other high-income countries, coverage focused on epidemiology of the evolving outbreak in these settings, often neglecting the longstanding and ongoing transmission of mpox in African countries. The relative media neglect of mpox in endemic countries reflected broader global health disparities; a minority of articles commented upon this media neglect, and later vaccine stockpiling:

“… there [has] been over 1,400 suspected cases of monkeypox this year in Africa and 66 deaths. It’s an unfortunate reflection of the world we live in that the international community is only now paying attention to monkeypox because it has appeared in high-income countries.”

(Tedros Ghebreyesus, quoted in The Sun, 09/06/2022)

“The UK government initially ordered 50,000 doses (the maximum, it says, that was immediately available), and a further 100,000 are expected to arrive in late September. This would be enough to treat 75,000 people.”

(The Guardian, 25/08/2022)

Articles published later in the outbreak reported on the decline in cases and uncertainties about the driver of reduced transmission. A common hypothesis across these articles was that while that vaccination was likely to have contributed to the reduction in cases, a significant behavioural shift within the gay and bisexual community was probably the major factor driving the fall (The Times, 03/10/2022).

Acknowledging the lack of familiarity with mpox among their readership, many articles described the natural history and pathogenesis of mpox, covering the incubation period, symptoms (and the difficulty distinguishing symptoms from other infections), and diagnosis:

“Early signs of the virus include a fever, headache, muscle aches, backache, swollen lymph nodes, chills and exhaustion…But its most unusual feature is a rash that often begins on the face, then spreads to other parts of the body, commonly the hands and feet.”

(Mail Online, 10/08/2022)

We identified a notable paucity of accounts of people with lived experience of mpox. In August 2022, a few articles featured Harun Tulunay, a sexual health and HIV advocate who had shared his experiences of mpox in an effort to normalise conversations about mpox and challenge the stigma that had rapidly emerged:

“I was scared I would die alone in my hospital room […] I’d never been in so much pain in my life.”

(Harun Tulunay (sexual health and HIV advocate) quoted in thesun.co.uk, 10/08/2022)

These rare personal accounts were often the only time the severity of pain and the consequences of isolation featured in coverage.

In response to the racist and stigmatising language attached to mpox during the 2022 outbreak, the WHO announced the change of nomenclature from “monkeypox” to “mpox” in November 2022. By this time, news media reporting on mpox in the UK was minimal precluding us from commenting on the adoption of the preferred term. However, our analysis sample did include coverage of discussions in the lead up to the name change:

“United States officials are believed to have put pressure on the World Health Organisation to change the name and warned they could act unilaterally if no action was taken. And now the decision to change the name of the virus to MPOX could be made on Wednesday…”

(mirror.co.uk, 23/11/2022)

### Delivery of health promotion

A key focus of UK news media reporting on mpox throughout 2022 was the delivery of health promotion messages. Coverage not only described official public health guidance but also shaped public perceptions of transmission, risk, and vaccination. Early reporting often emphasised tracing cases to identifiable sources and highlighted GBMSM as the most affected group. As the outbreak progressed, media narratives evolved to include more nuanced discussions around close-contact transmission, stigma, and barriers to vaccine access.

#### Public health messaging

News media relayed targeted public health messages from institutions such as the UK Health Security Agency (UKHSA), emphasising that “*monkeypox continues to be transmitted primarily in interconnected sexual networks*” of men who have sex with men (Mail Online, 10/09/2022). Coverage also included alerts issued by gay and bisexual-oriented dating apps warning users about mpox transmission (Mail Online, 24/05/2022).

Alongside these targeted messages, a number of articles highlighted the importance of broader and more inclusive communication to avoid perpetuating negative stereotypes. While some reports highlighted that anyone could contract mpox through close or intimate contact, others drew attention to concerns raised by international bodies about stigmatising portrayals:

“Experts have stressed that anyone can get monkeypox as it is spread by close or intimate contact, with the UN having warning that some media portrayals of Africans and LGBTQ+ people reinforce homophobic and racist stereotypes and exacerbate stigma.”

(The Guardian, 23/07/2022)

Information was also provided on what to do in the case of symptoms and/or a confirmed diagnosis. News media reported advice from the UKHSA to self-isolate (especially avoiding contact with children and/or pets) and to contact local sexual health services if individuals had symptoms such as skin lesions, and/or close contact with someone with suspected or confirmed mpox in order to *“limit the spread of this infection”* (Mail on Sunday, 24/05/22). Readers were informed that the UKHSA would contact individuals who had been in close contact with mpox and advise them further (Mail Online, 01/06/22).

#### Transmission

In the early stages of the outbreak, media narratives largely concentrated on trying to link cases to identifiable sources of transmission-particularly travel to Central and West Africa (or close household contact with someone who had travelled to the region):

“Eight days ago, the UK Health Security Agency (UKHSA) revealed that someone with ‘a recent travel history from Nigeria’ was being treated at Guy’s and St Thomas’ Hospital in Westminster.”

(Unnamed source at UKSHA, Mail on Sunday, 15/05/2022)

“Yesterday, it said the new cases were two people in the same household.”

(Unnamed source at UKHSA, Mail on Sunday, 15/05/2022)

As the outbreak progressed, some reporting attempted a more nuanced messaging, emphasising the role of close physical contact regardless of sexual orientation:

“By nature, sexual activity involves intimate contact, which one would expect to increase the likelihood of transmission, whatever a person’s sexual orientation and irrespective of the mode of transmission”

(Mike Skinner (virologist at Imperial College London), quoted in MailOnline, 24/05/2022)

#### Risk

Once close physical contact was identified as the primary mode of transmission, the dominant narrative framed the gay community as the population at highest risk of acquiring mpox in the UK and other high-income countries. This framing was reinforced by media coverage that linked transmission to specific events and behaviours:

“Two recent events for the gay community in Belgium and Spain may have acted as “superspreader” events, with the virus spreading during close contact such as sexual liaisons.”

(thetimes.co.uk, 24/05/2022).

Here we note the use of “superspreader”, a term that has no clear definition and has been critiqued for oversimplifying complex transmission dynamics and for assigning moral blame to individuals and/or groups [37]. This reporting represented mpox as a particular threat to communities of GBMSM, requiring vigilance, behaviour change, and prompt medical attention in the event of symptoms. GBMSM were urged to:

“temporarily consider reducing their number of sex partners or refrain from group or anonymous sex to reduce their risk of catching the virus.

(Unnamed source from the World Health Organization, quoted in Mail Online 20/09/2022)

Risk reduction advice from public health institutions was predominantly aimed at gay and bisexual men and this was reflected in news media coverage. For example, in the run-up to UK Pride celebrations, news media repeated advice from national sexual health organisations:

“We’re reminding everyone of the symptoms of monkeypox, and gay and bisexual men in particular, to be especially aware and seek advice immediately by calling NHS 111 or their local sexual health clinic if they have concerns.”

The Times, 01/06/2022)

#### Vaccination

The mpox vaccination programme was introduced in the UK in June 2022. By early August, only 14,227 [38] vaccines had been administered, although this figure had more than doubled to 30,734 by mid-August [38]. Initial reporting of the vaccination programme focused on providing background information on the vaccines available, how they worked, vaccination strategy, eligibility criteria, the cost and their potential to protect against mpox:

“The smallpox vaccine, called Imvanex in the UK and Jynneos in the US, can protect against monkeypox because the viruses behind the illnesses are closely related. The jab, thought to cost £20 per dose, contains a modified vaccinia virus, which is similar to both smallpox and monkeypox, but does not cause disease in people.”

(MailOnline, 24/05/2022)

Media coverage also described possible strategies for vaccine roll out:

“MailOnline revealed monkeypox patients and their close contacts, including NHS workers, are being offered the Imvanex smallpox vaccine. The strategy, known as ring vaccination, involves jabbing and monitoring anyone around an infected person to form a buffer of immune people to limit the spread of a disease.”

(MailOnline, 24/05/2022)

And later who was eligible for the targeted approach that was adopted:

“Once the vaccine centre was set up, regular patients who were eligible for the vaccine (those on pre-exposure prophylaxis medication (PrEP); HIV-positive patients; those who recently had STIs; men with multiple partners, who participate in group sex or attend “sex on premises” venues) were contacted”.

(The Guardian, 25/08/2022)

By early July 2022, however, news media attention had turned to highlighting overwhelming demand, particularly among GBMSM:

“We’re seeing up to 200 people a day, but we could see far more if we had supply.”

(Clinician at a large London sexual health service, quoted in The Guardian, 25/08/2022)

This soon escalated into reporting on the frustrations and difficulties individuals experienced accessing vaccination due to lack of information about where to obtain the vaccine, as well as appointment slots filling up quickly:

“I’ve been trying to get the vaccine for weeks… finding out how to protect myself has been almost impossible.”

(Clinician at a large London sexual health service, quoted in The Guardian, 25/08/2022)

Shortfalls in UK vaccine supply became a dominant focus over the summer of 2022, with reporting of concerns among UK sexual experts:

“By current estimates, only 8,300 vaccines remain available…, making it likely that the remaining number of vaccines will run out in approximately 10 to 20 days.”

(Claire Dewsnap, President of the British Association of Sexual Health and HIV, quoted in The Guardian, 10/08/2022)

In the context of limited supply, experts warned of the importance of prioritising vaccines for areas of the UK reporting the highest incidence:

“The very limited doses of vaccine we have left in the country must now be prioritised for those most at risk in the places with the highest reported cases. That means ensuring sexual health services in places like Brighton, Manchester and Essex are given provision alongside London.”

(Ian Green, Chief Executive of the Terrence Higgins Trust, quoted in MailOnline, 10/08/2022)

Just as mpox incidence was peaking, UK news media reflected community and professional concerns about difficulties accessing vaccination because of supply shortages and/or limited information. In response, articles began to feature criticisms of the government response from UK experts who identified lack of funding and procurement issues as key challenges, as well as reporting potential lack of political will to address a public health emergency affecting mainly gay men:

“‘If it ends up endemic in gay men, then so be it.’”

(An unnamed senior public health official, quoted in The Guardian, 25/08/2022)

The perceived slow governmental response to mpox, was contrasted with the rapid mobilisation seen during the COVID-19 pandemic, with clear parallels drawn between the response to mpox and the neglect of marginalised communities during the early years of the HIV pandemic.

“‘We saw what happened with Covid,’ he says. ‘Every resource possible was put towards ensuring the vaccine was available to those who needed it… With this, we’re not waiting for clinical trials, or breakthroughs in science.’ There is a vaccine... ‘Yet no agency or politician will even commit to providing access to everyone who wants a vaccine... Instead, this community is once again being placed at risk’”

(Greg Owen, a sexual health and HIV activist from the Terrence Higgins Trust, The Guardian, 25/08/2022)

### ‘Anchoring’ mpox to other infectious diseases

News media faced the challenge of rendering a new infectious disease threat familiar, drawing upon diseases that were likely to have been within readers’ frame of reference. The 2022 mpox outbreak occurred soon after the COVID-19 pandemic, making comparisons between the two both immediate and inevitable.

Mpox was largely framed as less of a threat than COVID-19 in terms of transmission:

“There’s no need to sound the alarm just yet. Monkeypox is not like COVID-19 - it’s nowhere near as transmissible.”

(The Guardian, 24/05/2022).

“Dr Kluge insisted the virus ‘will not require the same extensive population measures’ as Covid but said ‘significant and urgent’ action was needed to prevent more cases.”

(MailOnline, 01/06/2022)

The most common anchor in news reporting was HIV, with some articles describing how the mpox outbreak evoked memories of the HIV pandemic at an individual level:

“‘I know people older than me are freaking out – seeing young, otherwise healthy gay and bisexual men turning up with lesions all over their faces. That’s a huge trigger for those who lived through that (HIV) epidemic.’”

(Greg Owen, a sexual health and HIV activist from the Terrence Higgins Trust, The Guardian, 25/08/2022)

Among younger generations, mpox generated fears of a public health crisis affecting GBMSM on a par with HIV.

“‘Some of the younger ones have been very anxious,’ Lombardi adds. ‘They’ve inherited this history that’s alarming for them: something going around that nobody knows much about, that’s sexually transmitted. It’s scary for them.’”

(Kim Lombardi, clinical lead for the vaccination team, Central and North West London NHS Foundation Trust, The Guardian, 25/08/2022)

As previously discussed, UK news media representations of mpox linked the outbreak closely with GBMSM communities. However, at the same time, there was some acknowledgement of the risks of doing so, and the need to avoid the stigmatising narratives and moral blame that was characteristic of early reporting of HIV:

“‘We have to be careful here because this doesn’t mean MSM [men who have sex with men] are at fault at all. “And we definitely shouldn’t echo the ridiculously inaccurate hysteria of HIV being a ‘gay disease’’”.

(Ranj Singh, TV personality and doctor, quoted in thesun.co.uk, 24/5/2022)

### Differences in framing across newspapers

While we did not conduct a formal content or discourse analysis of positionality and tone, clear differences in framing emerged across the newspapers included in this study. The most marked contrasts were between MailOnline (and its associated print titles) and the Guardian. The Mail accounted for the largest number of articles (*n* = 29) and typically adopted a largely neutral, informational tone, foregrounding concern and providing frequent factual updates, particularly in the early stages. However, coverage often repeated sections of text referencing GBMSM, which may have reinforced the association between GBMSM and mpox despite explicit caution against stigma in the articles. By contrast, Guardian articles, while similarly grounded in factual reporting, more consistently adopted a critical stance towards perceived systemic failings and delays in the public health response, alongside stronger elements of empathy and advocacy for GBMSM and cautious optimism regarding vaccination.

## Discussion

In our analysis of reporting on mpox in five UK newspapers between May and December 2022, we found that coverage peaked at the start of the outbreak in May 2022 but declined rapidly from June onwards with no articles published in December 2022. This decline in media interest predated the decline in mpox cases in the UK, a trend also observed during the COVID-19 outbreak [14]. This early peak and subsequent waning of media interest as novelty diminishes has been reported in coverage of other infectious diseases, most notably HIV [39].

This observed lack of sustained media interest in mpox is concerning given the important role news media plays during outbreaks. Our thematic analysis revealed that news media helped introduce mpox to a readership who were unfamiliar with the disease, providing information on its origins, evolving epidemiology, natural history, pathogenesis and symptoms. Articles often “anchored” mpox to other infectious diseases such as COVID-19 and HIV, to “render this previously unfamiliar disease familiar” [25]. As the outbreak evolved, the focus of articles shifted to the delivery of health promotion, including transmission, management, and vaccination.

The role of news media in mobilising knowledge to a wide readership is well-recognised [40]. However, as highlighted by Vaughan and Power (2023) in their analysis of newspaper representations of HIV, beyond disseminating information, news media shapes public discourse and perceptions of infectious diseases [15]. Our analysis identifies numerous ways in which this occurred during the 2022 mpox outbreak. First, news media outlets selected which aspects of mpox to prioritise in its reporting, changing the focus over the course of the outbreak. Whereas initial reporting mainly covered communication of information on what mpox was, this rapidly gave way to public health messaging, and later critical engagement with discussions about global health, vaccine equity, and stereotyping. Second, news media reporting framed mpox as a disease predominantly affecting GBMSM, despite some acknowledgement of the risk of amplifying homophobia, as seen in HIV. The frequent linking of mpox with GBMSM communities, sometimes drawing upon stereotypes of sexual behaviour and neglecting the diversity of communities and sexual behaviours within this population, has been reported in a previous study, and may have contributed to mpox stigma [26]. Moreover, framing mpox discursively as a “gay disease” positioned the threat as limited to specific communities, potentially reinforcing stigma and diminishing the perceived need for broader public health action. This highlights the tension between reflecting the epidemiology of an evolving outbreak, and ensuring targeted messaging reaches those at highest risk, and causing harm to an already marginalised group. This dilemma echoes debates during the HIV epidemic, particularly in the mid-1990s, around what was described as the “de-gaying of AIDS” through an “equal opportunities virus” policy [41]. These policies were developed with the intention of reducing stigma by avoiding the association of HIV with gay men. However, they were heavily criticised for diverting attention and resources away from those most affected, especially GBMSM, thereby undermining targeted prevention and care efforts [41].

During global outbreaks, news media commonly draw on expert opinion as a source of scientific knowledge and advice to communicate clear and reliable information [20]. Articles in this analysis cited a diverse range of sources, including national governmental public health agencies, including UKHSA and the US Centers for Disease Control and Prevention, as well as regional and global agencies such as the European Centre for Disease Prevention and Control and the World Health Organization. Additional sources included pharmaceutical companies, academics, non-governmental community organisations, and clinicians. This use of a diverse range of sources reflects longer-term changes in health reporting identified by Hallin et al. [40]. Similarly, reporting in our study often combined multiple expert voices within a single article and used expert statements to provide factual updates, seemingly to strengthen the authority of coverage. Many experts were unnamed or identified by institutional affiliation; those who were named were predominantly based in London and worked within government agencies, charities, or NHS services, with UKHSA staff featuring particularly prominently. This concentration of named expert sources in London aligned with the geographical distribution of cases during the outbreak, with approximately 70% of mpox cases in 2022 reported in the capital [42].

Social media has disrupted traditional print media, rapidly becoming a primary source of news and changing how information is generated and communicated [24, 25]. Studies have explored the role of social media in the 2022 mpox outbreak, primarily focusing on the role of Twitter/X in public health messaging and in propagating mis/disinformation [43, 44]. Compared to social media, news media, with its longer format and more time to produce content, can offer in-depth reporting and expert commentary, both of which are critical for conveying complex public health issues in a comprehensive and accessible way [45]. News media therefore remains a crucial partner in health messaging during infectious disease outbreaks.

We identified some missed opportunities in news media reporting. First, there was a notable paucity of personal testimonies of individuals with mpox, in contrast to the sharing of lived experiences of mpox on social media [43]. On these platforms, individuals were able to describe their symptoms and experience of healthcare, offer advice to others, and contribute to normalising discussions about mpox [43]. Of course, some platforms (such as those using video) may be especially well suited to the sharing of personal testimonies. However, in ignoring personal accounts, newspaper articles failed to articulate the level of physical and mental distress experienced by people who had acquired mpox [5, 46].

Second, while the discursive framing of mpox as being linked to GBMSM may have reflected the epidemiology, it may have also perpetuated negative stereotypes. It is important for news media to be aware of these risks and to consider how it can minimise harm to already marginalised communities. One way of doing so is by incorporating personal testimonies from those directly affected, which can serve to humanise the outbreak, challenge reductive or stigmatising narratives, and highlight the diversity of experiences within GBMSM communities.

Another opportunity lies in the greater use of non-governmental and community-based organisations to help shape and amplify outbreak communication-particularly in this case organisations serving GBMSM. While such organisations did appear in news coverage, this tended to occur later in the outbreak, most notably in relation to vaccination, with a small range of organisations represented and limited coverage. These organisations play a crucial function in interpreting public health guidance, communicating practical information about vaccination, and countering stigmatising narratives, as also reflected in our linked focus group study [47]. Their importance underscores the need for outbreak communication strategies to be co-developed with community-based organisations and supported by coordinated communication structures that formally embed them alongside public health agencies, clinicians, academics and journalists, such as the national STI outbreak communication group currently being developed within the NIHR Health Protection Research Unit in Sexual Health and Blood Borne Infections at UCL [48].

Several limitations should be acknowledged. The analysis was restricted to five mainstream UK news outlets and did not include other potentially influential sources, such as LGBTQ + or community-specific press, which may have played a significant role in shaping public understanding within GBMSM communities. Although this represents a limitation, mainstream news outlets nonetheless have the potential to shape wider public understanding, particularly among those not directly engaged in GBMSM networks. The search strategy was limited to headlines containing “mpox/monkeypox/monkey pox,” to retrieve a focused and manageable sample of articles where the outbreak was the primary subject. This is similar to a strategies used by other authors [25]. However, limiting to these terms may have excluded more indirect or potentially stigmatising coverage that did not explicitly name the virus, particularly where sensationalist or coded language was used. Furthermore, only a sample of identified articles was analysed; however, this was mitigated through a random sampling approach to minimise selection bias. Formats, such as podcasts and video content, which are increasingly central to how news is consumed, were also excluded. The focus on English-language reporting within the UK further limits the generalisability of findings to other linguistic or cultural contexts. Finally, while this paper provides an overview of key themes in news media coverage, a more detailed linguistic analysis of reporting is presented in a separate manuscript submitted elsewhere [49].

However, this analysis also has a number of strengths. It systematically captures how the UK news media framed and communicated information about the 2022 mpox outbreak. By selecting a mix of tabloid and broadsheet newspapers across the political spectrum, the study covers a broad and balanced view of media narratives. It also highlights how coverage evolved over time, identifying important patterns such as initial media attention followed by a decline in interest, despite rising case numbers. In addition, the involvement of a community representative as a co-author helped ground the analysis in lived experience and ensured that interpretations were informed by community perspectives, adding relevance to the work.

## Conclusion

UK news media interest in mpox peaked early in the outbreak but declined as case numbers rose. Whilst the media played a crucial role in disseminating information and public health messages, its framing of GBMSM may have contributed to reinforcing stigma. Our analysis underscores the need for coordinated outbreak communication strategies that engage key stakeholders, and including both print and social media, as part of preparedness efforts. The lack of personal testimonies represents a missed opportunity for more inclusive and relatable messaging. We recommend that future outbreak reporting actively involves affected communities to ensure communication is both effective and non-stigmatising for those most impacted. In addition, our findings highlight the importance of training professionals across academic, charity, and health sectors, as well as community advisory board members, in media communication, to promote accurate and stigma-free narratives during health crises.

## Supplementary Information

Additional file 1. Table of analysis themes, sub-themes and codes (pdf). This file provides a table detailing the analysis themes, sub-themes and codes reported in this paper.

## Data Availability

The dataset analysed during the current study are available in the public domain and in the NexisUK repository (https:/www.lexisnexis.com/en-gb/products/research-insights/nexis).

## Abbreviations

CAB: Community advisory board
DRC: Democratic Republic of the Congo
GBMSM: Gay, bisexual, and other men who have sex with men
MVA-BV: Modified vaccinia Ankara- Bavarian Nordic
PHEIC: Public health emergency of international concern
UKHSA: UK Health Security Agency
UK: United Kingdom
WHO: World Health Organization
