# Supplementary material for: Representations of mpox in UK news media during the 2022 outbreak: a qualitative analysis
Source: BMC Public Health. 2026 Jun 19;26:2355. doi: 10.1186/s12889-026-28060-2 (PMC13471181; doi:10.1186/s12889-026-28060-2)
Supplement: Supplementary file 1 [file 12889_2026_28060_MOESM1_ESM.pdf]

Additional File 1: Table of analysis themes, sub-themes and codes

Table 1: Table of analysis themes, sub-themes and codes

**Theme**	**Sub-theme**	**Codes**
Mpox characteristics	Natural history and pathogenesis	diagnosis information, disease outcomes, disease outcomes in children, strain of clade, incubation period, symptom description, treatment
	Epidemiology	number of cases, decline in cases, situation in the UK and other high-income countries, current situation in Africa
	Origins	lab monkeys, first human cases, first identified in West Africa, geographical history, animal transmission, GBMSM festivals and events
	Global emergency	global public health response, renaming monkeypox
Delivery of health promotion	Public health messaging	disease management and advice {advice about contagiousness, advice to isolate, be alert for symptoms, contact tracing, health seeking advice, risk reduction advice, hard to get advice} sexual health as first line service, public health and health response
	Risk	anyone can get mpox, men having casual sex at risk, multiple sexual partners, population affected are gay men, risk of mpox becoming endemic, symptoms minimised as no death
	Transmission	animal or bushmeat transmission, community transmission, child transmission, respiratory transmission, sexual transmission & sexual networks, transmission through human-to-human close contact
	Vaccination	global vaccine access and demand, running out of vaccine, second dose of vaccine, smallpox vaccination as protection, vaccination access and demand, vaccination effectiveness, vaccine allocation – global equity, vaccine allocation - UK
Anchoring to other infectious diseases	Comparison to COVID-19	No codes were identified.
	Comparisons to HIV	
	Comparisons to smallpox	
